# Two-dimensional patterns in neural fields subject to finite transmission speed

**DOI:** 10.1186/1471-2202-15-S1-P16

**Published:** 2014-07-21

**Authors:** Eric Nichols, Kevin Green, Axel Hutt, Lennaert van Veen

**Affiliations:** 1 INRIA Nancy, Team NeuroSys, 615 rue du Jardin Botanique, 54600 Villers-lès-Nancy, France; 2 Faculty of Science, University of Ontario Institute of Technology, 2000 Simcoe Street North, Oshawa, L1H 7K4 Ontario, Canada

## 

This work analyzes and implements finite axonal transmission speeds in two-dimensional neural populations. The biological significance of this is found in the rate of spatiotemporal change in voltage across neuronal tissue, which can be attributed to phenomena such as delays in spike propagation within axons, neurotransmitter activation and the time courses of neuron polarization and refraction. The authors build upon the finite transmission speed work in [1].

Linear analysis about a spatially homogeneous resting state of the neural population dynamics is performed. The analyses of the resulting analytical expressions guide the parameter selection for simulations. For simulation, computation of the transmission-delayed convolution between the kernel and firing rates is performed with a fast Fourier transform as in [1].

The Neural Field Simulator [2] is used to simulate the activity of the field. We extended the simulator by the implementation of a large class of kernels reflecting global excitation, global inhibition, local excitation-lateral inhibition and local inhibition-lateral excitation. Moreover, we extended the tools by an automatic root finder to compute stationary states and an automatic root finder of the characteristic equation of the linear dynamics. These latter features facilitate the user to perform the linear analysis. Further adjustments to the simulator include a provision to modify neural field variables online while simulations are ongoing and three-dimensional displays of disparate parts of the neural field, such as the external input, kernel and firing rate.

**Figure 1:**
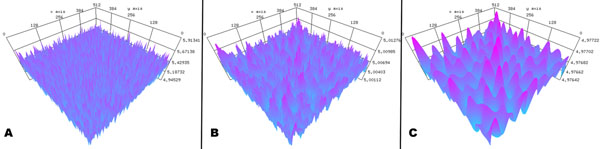
A neural field simulation. A noisy field **A** evolving **B** into a Turing pattern **C**.

We find Turing patterns appear when starting the simulations with the derived conditions for stationary instability. This is shown in Figure 1(A,B,C). Simulations with travelling wave patterns are also performed using parameter sets for the non-stationary instabilities, and the effects of finite transmission speeds are analyzed and visualized. The software tool provides a large set of analysis and visualization tools, that promises to speed up the analysis of two-dimensional neural field dynamics and hence accelerates research on neural population dynamics.
